# Droplet Digital PCR for *BCR–ABL1* Monitoring in Diagnostic Routine: Ready to Start?

**DOI:** 10.3390/cancers13215470

**Published:** 2021-10-30

**Authors:** Maria Teresa Bochicchio, Jessica Petiti, Paola Berchialla, Barbara Izzo, Emilia Giugliano, Emanuela Ottaviani, Santa Errichiello, Giovanna Rege-Cambrin, Claudia Venturi, Luigiana Luciano, Filomena Daraio, Daniele Calistri, Gianantonio Rosti, Giuseppe Saglio, Giovanni Martinelli, Fabrizio Pane, Daniela Cilloni, Enrico M. Gottardi, Carmen Fava

**Affiliations:** 1 Biosciences Laboratory, IRCCS Istituto Scientifico Romagnolo per lo Studio e la Cura dei Tumori (IRST) “Dino Amadori”, 47014 Meldola, Italy; teresa.bochicchio@irst.emr.it (M.T.B.); daniele.calistri@irst.emr.it (D.C.); 2 Department of Clinical and Biological Sciences, University of Turin, AOU San Luigi Gonzaga, Orbassano, 10043 Turin, Italy; jessica.petiti@unito.it (J.P.); filodaraio@tiscali.it (F.D.); giuseppe.saglio@unito.it (G.S.); daniela.cilloni@unito.it (D.C.); carmen.fava@unito.it (C.F.); 3 Department of Molecular Medicine and Biotechnology, University Federico II & Ceinge Advanced Biotechnologies Center, 80138 Naples, Italy; barbara.izzo@unina.it; 4 Division of Internal Medicine and Hematology, San Luigi Gonzaga Hospital, Orbassano, 10043 Turin, Italy; e.giugliano@sanluigi.piemonte.it (E.G.); giovanna.rege@libero.it (G.R.-C.); enricogottardi@libero.it (E.M.G.); 5 Istituto di Ematologia “Seràgnoli”, IRCCS Azienda Ospedaliero-Universitaria di Bologna, 40138 Bologna, Italy; emanuela.ottaviani@aosp.bo.it (E.O.); claudia.venturi7@unibo.it (C.V.); 6 CEINGE Advanced Biotechnologies, 80131 Naples, Italy; errichiello@ceinge.unina.it; 7 Hematology Unit “Federico II Hospital”, University of Naples, 80131 Naples, Italy; lulucian@unina.it; 8 IRCCS Istituto Scientifico Romagnolo per lo Studio e la Cura dei Tumori (IRST) “Dino Amadori”, 47014 Meldola, Italy; gianantonio.rosti@irst.emr.it (G.R.); giovanni.martinelli@irst.emr.it (G.M.); 9 Department of Clinical Medicine and Surgery, University Federico Secondo, 80138 Naples, Italy; fabrizio.pane@unina.it

**Keywords:** chronic myeloid leukemia, ddPCR, *BCR–ABL1*, deep molecular response, treatment-free remission

## Abstract

**Simple Summary:**

The introduction to clinical practice of a treatment-free remission approach in chronic myeloid leukemia patients with a stable deep molecular response highlighted how crucial it is to monitor the molecular levels of *BCR–ABL1* as accurately and precisely as possible. In this context, the droplet digital PCR (ddPCR) presents an alternative methodology for such quantification. To hypothesize the introduction of this technology in routine practice, we performed a multicentric study that compares ddPCR with the standard methodology currently used. Our results demonstrate that the use of ddPCR in clinical practice is feasible and could be beneficial.

**Abstract:**

*BCR–ABL1* mRNA levels represent the key molecular marker for the evaluation of minimal residual disease (MRD) in chronic myeloid leukemia (CML) patients and real-time quantitative PCR (RT-qPCR) is currently the standard method to monitor it. In the era of tyrosine kinase inhibitors (TKIs) discontinuation, droplet digital PCR (ddPCR) has emerged to provide a more precise detection of MRD. To hypothesize the use of ddPCR in clinical practice, we designed a multicentric study to evaluate the potential value of ddPCR in the diagnostic routine. Thirty-seven RNA samples from CML patients and five from healthy donors were analyzed using both ddPCR QXDx^TM^ *BCR-ABL* %IS Kit and LabNet-approved RT-qPCR methodologies in three different Italian laboratories. Our results show that ddPCR has a good agreement with RT-qPCR, but it is more precise to quantify *BCR–ABL1* transcript levels. Furthermore, we did not find differences between duplicate or quadruplicate analysis in terms of *BCR–ABL1*% IS values. Droplet digital PCR could be confidently introduced into the diagnostic routine as a complement to the RT-qPCR.

## 1. Introduction

Chronic myeloid leukemia (CML) is a clonal hematopoietic disorder characterized by increased and unregulated growth of differentiated myeloid cells that accumulate in the blood [1]. The reciprocal translocation between the long arms of chromosome 9 and 22 t(9;22)(q34;q11) leads to the formation of the *BCR–ABL1* fusion gene encoding for the homonym protein with hyper tyrosine kinase activity. The *BCR–ABL1* rearrangement occurs in about 95% of CML patients with two major (e13a2 and e14a2), one minor (e1a2), and some rare variants [2]. During diagnosis, a qualitative PCR on a peripheral blood sample is mandatory to identify the specific *BCR–ABL1* isoform while real-time quantitative polymerase chain reaction (RT-qPCR) in necessary to assess the response to tyrosine kinase inhibitors (TKIs) therapy [3] since the majority of patients taking TKIs obtain a major molecular response (MMR) and about one third to one fourth achieve a deep molecular response (DMR) with fewer detectable leukemic cells [2].

For this reason, *BCR–ABL1* mRNA levels represent the key molecular marker for the evaluation of minimal residual disease (MRD), defining the depth of molecular remission and guiding clinical decisions such as a change in TKI or, more recently, discontinuation of therapy [4]. The criteria for the monitoring of *BCR–ABL1* follow the recommendation described by the Europe Against Cancer (EAC) program and successively revised especially for the DMR scoring [5,6]. In addition to the EUropean Treatment Outcome Study (EUTOS) consortium, the Italian laboratory network LabNet (i) is actively involved in the standardization of *mBCR–ABL1* quantification and (ii) properly coordinates quality control rounds in order to monitor and eventually improve the network laboratories’ performances. The first point has been improved over the years thanks to the introduction of the use of a laboratory-specific conversion factor [7]. Furthermore, in an era in which DMR is becoming the target to be achieved in common clinical practice and represents the mandatory prerequisite for a treatment-free remission approach [3], a highly sensitive and accurate technique for *BCR–ABL1* levels monitoring is necessary. In this context, droplet digital PCR (ddPCR) represents an innovative evolution of RT-qPCR with many practical advantages. This technology is based on a massive partition of the sample into thousands of droplets in which single end-point PCR reactions are performed. Being independent of calibrators and standard reference curves [8,9] and overcoming the RT-qPCR limitations in counting the e13a2 variant [10], ddPCR appears to be the most reliable and reproducible method for MRD monitoring compared to RT-qPCR. With the introduction of MR4 and MR4.5 levels of molecular response (MR) in CML patients, the use of ddPCR seems to be very attractive [11,12,13,14]. To hypothesize the introduction of this technology in clinical practice, we designed a multicentric study that evaluated the potential value of ddPCR in the diagnostic routine. In particular, we compared ddPCR QXDx™ BCR-ABL %IS Kit (Bio-Rad, Hercules, CA, USA) with LabNet-approved RT-qPCR methodologies in monitoring *BCR–ABL1* levels in CML patients by assessing the agreement between the measures obtained, the repeatability (intra-laboratory variability), and the reproducibility (inter-laboratory variability) of the two methods. Furthermore, we investigated the Limit of Blank (LoB) of ddPCR QXDx™ BCR-ABL %IS Kit in our cohort of CML samples.

## 2. Materials and Methods

### 2.1. Samples Collection and Processing

Peripheral blood (PB) leukocytes were isolated by Buffy Coat procedure from 37 CML patients, according to LabNet recommendations, and 5 healthy donors. Total RNA was extracted using Maxwell 16 LEV simplyRNA Blood kit (Promega, Madison, WI, USA), following the manufacturer’s instructions. RNA samples were then quantified by NanoDrop™ One/OneC Microvolume UV-Vis Spectrophotometer (Thermo Fisher Scientific, Waltham, MA, USA) and aliquoted for RT-qPCR and ddPCR experiments. CML samples were divided into 5 groups based on the MR levels [3,6] obtained in routine RT-qPCR analysis as follows: group 1, MR < 3, *n* = 10; group 2, MR 3, *n* = 9; group 3, MR 4, *n* = 8; group 4, MR 4.5 and 5, *n* = 10, and group 5, healthy donors, *n* = 5.

### 2.2. RT-qPCR for BCR–ABL1 p210 Quantification

*BCR–ABL1* p210 was quantified by RT-qPCR in 3 different Italian laboratories, namely: Lab 1 (Lab of Internal Medicine specialized in Hematology, San Luigi Gonzaga Hospital, Orbassano, Turin, Italy), Lab 2 (Lab of Molecular Biology “L. & A. Seràgnoli”, Sant’Orsola-Malpighi Hospital, Bologna, Italy), and Lab 3 (Lab of Oncological Hematology, CEINGE Advanced Biotechnology, Naples, Italy). Lab 2 and 3 performed 1 amplification session each, while Lab 1 performed 3 independent sessions. *BCR–ABL1* p210 was quantified with three different methods: by using the One-Step BCR-ABL P210 ELITe MGB Kit (ELITech Group, Turin, Italy), according to manufacturer’s protocol (Lab 1); by using the One-Step Philadelphia SensiQuant Kit (Bioclarma, Turin, Italy), according to the manufacturer’s instructions (Lab 2); and as described by Gabert et al. [15] (Lab 3). The results obtained were corrected for the laboratory-specific conversion factor, as recommended by the Standard Operating Procedures of the Labnet CML network (GIMEMA group). The target quantification for each sample was expressed in the International Scale (IS) as *BCR–ABL1/ABL1* %IS.

### 2.3. ddPCR for BCR–ABL1 p210 Quantification

*BCR–ABL1* p210 was also quantified by ddPCR. Lab 2 and 3 performed 1 amplification session each, while Lab 1 performed 3 independent sessions. The *BCR–ABL1* fusion and *ABL1* transcripts were tested in multiplex in the same well. All ddPCR experiments were performed using QXDx™ BCR-ABL %IS Kit (Bio-Rad, Hercules, CA, USA) on the QX200 system (Bio-Rad), according to the manufacturer’s instructions. Samples were tested in quadruplicate. Each replicate was partitioned into about 20,000 droplets by a droplet generator and amplified by PCR. Plates were then loaded onto the QX200 droplet reader (Bio-Rad) and analyzed by QuantaSoft^TM^ software (version 1.7.4, Bio-Rad). The target quantification for each sample was expressed as *BCR–ABL1/ABL1* %IS.

### 2.4. Statistical Analysis

The Bland–Altman analysis was performed and the bias with the 95% Limits of Agreement (95% LoA), the measurement errors, and the coefficients of reproducibility and repeatability were reported for the measurement of the agreement between the two techniques. Furthermore, the Limit of Blank (LoB) was evaluated for the Kit QXDx^TM^ BCR-ABL %IS. All analyses were stratified by the level of disease.

The bias was estimated by the mean difference between the measurements of the two methods. An LoA of 95% represents the prediction limits of the differences between the methods and was estimated as *d* ± 1.96 × *s*, where *d* and *s* are the mean and the standard deviation of the differences, respectively. Only data from Lab 1 were considered. The coefficient of repeatability is defined as the upper limits of a prediction interval for the absolute difference between two measurements by the same method on the same item under identical circumstances (same laboratory, same operator).

The coefficient of reproducibility is defined as the upper limits of a prediction interval for the absolute difference between two measurements by the same method on the same item under different circumstances, normally referring to different laboratories. To estimate measurement errors, coefficients of repeatability, and reproducibility, a variance component model was fitted with replicate measurements in each method by item stratum. Restricted-Maximum Likelihood (REML) estimates of the relevant variance component were returned. For the coefficient of repeatability, only data from Lab 1, with replicates, were considered. For the coefficient of reproducibility, data from Lab 1, without replicates, Lab 2, and Lab 3 were considered. Healthy donors were excluded.

## 3. Results

### 3.1. Comparison between RT-qPCR and ddPCR Results

To compare the agreement between the two methods, we considered data obtained in Lab 1 in three independent sessions with the same experimental conditions. A total of 252 measurements were included in this analysis, 126 for the ddPCR and 126 for the RT-qPCR, divided as follows: 60 for group 1, 54 for group 2, 48 for group 3, 60 for group 4, and 30 for group 5. A Student’s *t*-test did not reveal significant differences between the mean values of *BCR–ABL1* obtained with the two techniques (Table 1).

Bland–Altman analysis was carried out to assess the agreement between ddPCR and RT-qPCR values (Table 2). The Bland–Altman analysis indicated that the bias, i.e., the difference between the *BCR–ABL1* values, is negligible in all levels of disease.

Several components contributed to the observed variability (the width of the 95% LoA), including the heterogeneity of the different samples, the replicates on the same sample, and the intrinsic variability of the methods. If we isolate the latter component, we notice that the measurement error is higher when using RT-qPCR for all levels of disease (Table 2).

To assess the intra-laboratory variability, we calculated the coefficient of repeatability of ddPCR and RT-qPCR on data obtained in Lab 1 in three independent sessions with the same experimental conditions. Results are reported in Table 3, showing that the coefficients of repeatability of ddPCR were smaller than RT-qPCR across all the levels of disease.

To assess the inter-laboratory reproducibility, we compared ddPCR and RT-qPCR results obtained in all the laboratories, without replicates. A total of 252 measurements were included in this analysis, 126 for the ddPCR and 126 for the RT-qPCR, divided as follows: 60 for group 1, 54 for group 2, 48 for group 3, 60 for group 4, and 30 for group 5. We calculated the reproducibility coefficient and we observed that the values obtained with ddPCR were smaller than qRT-PCR for all the levels of disease (Table 4).

### 3.2. ddPCR: Differences between Duplicates and Quadruplicates

**Figure 1:**
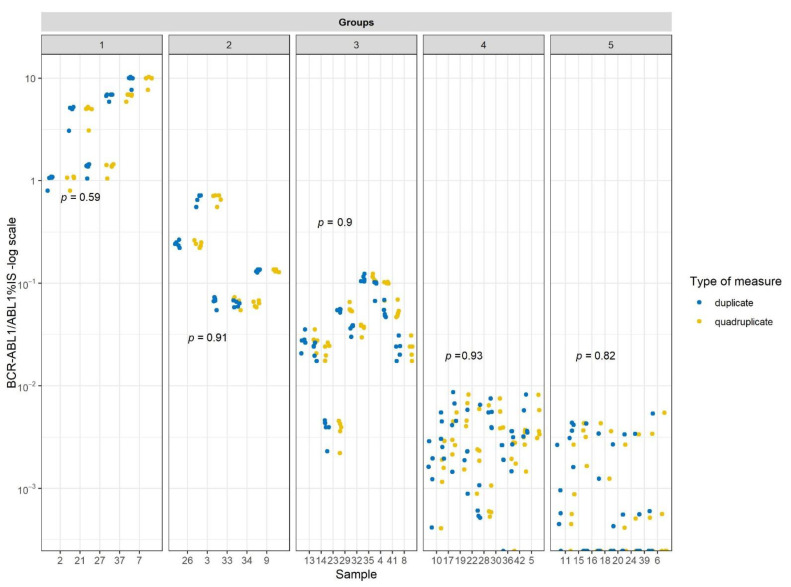
*BCR–ABL1/ABL1* %IS values obtained from duplicate (blue) or quadruplicate (yellow) analysis for all the levels of disease.

Since RT-qPCR is carried out in duplicate according to recommendation, we analyzed the differences between the results obtained by performing ddPCR analysis in quadruplicate or duplicate for all the levels of disease. Since the samples were tested in quadruplicate in our experiments, we randomly chose two replicates for the comparison. No differences between the *BCR–ABL1/ABL1* %IS values obtained from duplicate or quadruplicate analysis for all the levels of disease were observed (Figure 1). A Wilcoxon signed-rank test for pairwise clustered data was carried out to test differences between duplicates and quadruplicates [16]. The Holm method for *p*-value adjustment was considered. No significant differences were found.

### 3.3. ddPCR: Estimation of Limit of Blank

To estimate the Limit of Blank (LoB) of the ddPCR BioRad assay, we considered results obtained in Lab 1 for *BCR–ABL1/ABL1* %IS in healthy donors (group 5, *n* = 5). The three independent runs performed in Lab 1 returned 15 *BCR–ABL1/ABL1* %IS values (quadruplicate analysis) calculated from 60 replicates. Among five samples from healthy subjects, we found four samples with a detectable analyte concentration in one out of four replicates. LoB was determined by calculating the 95th percentile of the blank values using a quantile multilevel regression model to take into account the three independent runs of each sample.

If we considered four positive samples out of 15 measurements—defining a sample as positive when at least one out of four replicates had detectable analyte concentration—then we obtained a LoB of 0.0007 *BCR–ABL1/ABL1* %IS. Instead, we obtained a LoB of 0.002, considering four positive replicates out of 60.

## 4. Discussion

Droplet digital PCR represents an innovative evolution of RT-qPCR. According to the “divide et impera” principle, each sample is partitioned into several thousand (up to 20,000) single end-point PCR reactions and is not affected by the inhibitors present, primer efficiency, or calibrators, nor does it require standard reference curves. The last two issues allow to overcome the RT-qPCR limitations in monitoring several fusion transcripts, such as both canonical and atypical *BCR–ABL1* breakpoints in CML patients [16] and *PML/RARA* in acute promyelocytic leukemia (APL) [17], guiding the therapeutic strategy. Regarding *BCR–ABL1* canonical transcripts, a variant-specific discrepancy in the evaluation of *BCR–ABL1* e13a2 and e14a2 mRNA was observed when EAC assay was used in RT-qPCR. In 2019, Bernardi et al. demonstrated that ddPCR can correctly quantify both variants, confirming that the ddPCR results are less affected than RT-qPCR by primer efficiency [10]. The recent possibility of undertaking a treatment-free remission (TFR) approach in patients with a stable DMR has underlined the importance of reliable and precise monitoring of *BCR–ABL1* molecular levels in CML patients treated with TKIs. In our experiments, we used a multiplex ddPCR assay for p210 *BCR–ABL1* Major translocations in the peripheral blood of CML patients, which can quantify the target (*BCR–ABL1* Major translocations) and the housekeeping (*ABL1*) genes in the same well, returning an absolute quantification. The results obtained are expressed as a percentage of *BCR–ABL1/ABL1* in the International Scale (IS) (https://www.bio-rad.com/sites/default/files/webroot/web/pdf/lsr/literature/12006672.pdf; last access: 31 August 2021). Indeed, each kit contains two IS calibrator checks, traceable to the First WHO international Genetic Reference Panel (09/138) [18], that allow expressing results as IS thanks to a kit-specific conversion factor. In the present article, we compared ddPCR and RT-qPCR in terms of the agreement, intra- and inter-laboratory reproducibility, differences between duplicate and quadruplicate tests, and Limit of Blank (LoB). According to previous studies [9,19,20], we demonstrated that ddPCR could be confidently introduced into the diagnostic routine as a complement to the RT-qPCR. Indeed, the results obtained in Lab 1 in three independent sessions with the same experimental conditions indicate that *BCR–ABL1/ABL1* %IS values determined by ddPCR and RT-qPCR are superimposable. Although we were able to demonstrate that ddPCR has a good agreement with RT-qPCR, ddPCR resulted in greater precision (higher repeatability) than RT-qPCR for all the levels of disease. Moreover, the ddPCR was found to be more reproducible than RT-qPCR in all the disease levels, meeting the efforts made by the CML community to standardize the results [5,7,15]. The improved reproducibility and repeatability of ddPCR compared to RT-qPCR which we observed, may partly explain the reason for the superiority of ddPCR in predicting relapses in TFR patients [14,21,22,23,24,25]. In the context of the imatinib suspension and validation (ISAV) study (NCT01578213), ddPCR was able to detect one *BCR–ABL1* positive cell out of 10^7^ cells, predicting relapse in CML patients after imatinib discontinuation [13]. Moreover, Fluidigm digital PCR assay has been used in the ENESTnext (NCT01227577) study, confirming that ddPCR is more sensitive than RT-qPCR in patients with confirmed MR4.5, since about 40% of analyzed samples had a detectable *BCR–ABL1* [26]. Our data also corroborated results previously obtained by Della Starza et al. in pediatric acute lymphoblastic leukemia, where they identify ddPCR as more accurate than RT-qPCR in the measurement of MRD, particularly in the advanced follow-up [27]. Moreover, we showed no differences between duplicate or quadruplicate analysis, even for the lowest levels of disease. This results in the possibility to routinely test each sample in duplicate, reducing laboratory time and costs. In addition, to make CML diagnostics as comparable as possible, a network consisting of several laboratories across both Europe and Italy (LabNet) was created. These laboratories undergo annual RT-qPCR rounds that allow the calculation of their laboratory-specific conversion factor [6,28]. This procedure is expensive and time-consuming for the participating laboratories. The use of the ddPCR could allow us to skip this step, while maintaining the ability to express the results in IS.

Finally, data obtained about LoB underline how it is necessary to identify guidelines for the interpretation of the positivity of a sample and, therefore, to define a cut-off to distinguish a sample as true-positive or true-negative. An example comes from the MRD monitoring of other hematological diseases, such as multiple myeloma, mantle cell lymphoma, and follicular lymphoma [29,30], where criteria to define when a positive signal could not be quantified were identified starting from the guidelines used for RT-qPCR analysis [31]. Further studies are needed to identify the correct method of interpreting the data.

## 5. Conclusions

Our data indicated a good correlation between the *BCR–ABL1/ABL1* results obtained by ddPCR and RT-qPCR. The performance of the ddPCR assay makes it a promising tool for routine MRD monitoring in CML patients.

## Figures and Tables

**Table 1:** Comparison of the mean values between ddPCR and RT-qPCR divided by group of disease level.

Group	*N* SAMPLES	*N* MEASUREMENTS	ddPCR (Mean (sd); Min–Max)	RT-qPCR (Mean (sd); Min–Max)	*p* ^1^
Group 1	10	60	2.589 (3.529) 0.055–9.993	2.620 (3.626) 0.087–11.425	0.880
Group 2	9	54	0.0490 (0.0395) 0.0039–0.1235	0.0747 (0.0410) 0.0094–0.1375	0.145
Group 3	8	48	0.00343 (0.00259) 0.00089–0.00869	0.00756 (0.00789) 0.00000–0.02070	0.527
Group 4	10	60	0.001 (0.002) 0.000–0.005	0.001 (0.005) 0.000–0.014	0.2
Group 5	5	30	0 (0) 0.000–0.001	0 (0) 0–0	0.317

^1^ Student *t*-test.

**Table 2:** Agreement between ddPCR and RT-qPCR methods.

Level of Disease	Bias		Measurement Error	
Group	RT-qPCR-ddPCR	(95%LoA)	RT-qPCR	ddPCR
Group 1	0.036	(−1.916; 1.989)	0.374	0.059
Group 2	0.028	(−0.016; 0.072)	0.015	0.004
Group 3	0.005	(−0.007; 0.017)	0.005	0.0000002
Group 4–5	0.001	(−0.006; 0.008)	0.003	0.001

**Table 3:** Precision.

Level of Disease	Coefficient of Repeatability	
Group	RT-qPCR	ddPCR
Group 1	1.059	0.168
Group 2	0.043	0.012
Group 3	0.015	0.0000004
Group 4	0.009	0.002

**Table 4:** Reproducibility.

Level of Disease	Coefficient of Reproducibility	
Group	RT-qPCR	ddPCR
Group 1	7.238	1.2820
Group 2	0.060	0.019
Group 3	0.013	0.0031
Group 4	0.007	0.004

## Data Availability

The data presented in this study are available on request from the corresponding author.
